# Triple Negative Breast Cancer With Choroidal Metastasis Responsive to Sacituzumab Govitecan and Radiation Therapy

**DOI:** 10.1002/cnr2.70462

**Published:** 2026-02-06

**Authors:** Nellie Nafissi, Aditya Mahadevan, Elaine Chiao, Nejina Rijal, Ritesh Parajuli

**Affiliations:** ^1^ Division of Hematology/Oncology University of California Irvine Health Orange California USA; ^2^ Department of Medicine University of California San Francisco San Francisco California USA; ^3^ Department of Medicine University of California Los Angeles Los Angeles California USA

## Abstract

**Background:**

Orbital metastases are rare in patients with breast cancer. However, medical management of orbital metastases is limited by the inability of treatment options to penetrate the blood‐brain barrier. In patients with triple negative breast cancer (TNBC), these treatment options are further limited. Sacituzumab govitecan, an antibody‐drug conjugate, has emerged as a promising agent for metastatic TNBC. However, to date, patients with central nervous system (CNS) disease have been excluded from corresponding clinical trials, making the efficacy of sacituzumab govitecan in patients with orbital metastases unclear.

**Case:**

A 61‐year‐old female was initially diagnosed with a left breast hormone receptor positive invasive ductal carcinoma, receiving neoadjuvant chemotherapy with doxorubicin, cyclophosphamide, and docetaxel and a partial mastectomy. Several years later, the patient presented with a cough and was subsequently diagnosed with metastatic triple negative breast cancer with hepatic, osseous, and right supraclavicular and thoracic nodal metastases. Concurrently, the patient noted floaters in her vision, later found to be consistent with orbital metastases. The patient received radiation therapy to both eyes and was started on Sacituzumab govitecan. Following cycle 1, the ophthalmic exam showed a dramatic decrease in the size of choroidal metastases.

**Conclusion:**

This case report documents the first case of orbital metastases successfully treated with radiation therapy and sacituzumab govitecan. As such, this case highlights the importance of sacituzumab govitecan as a potentially effective option for TNBC patients with CNS disease. Further studies and real‐world data are needed to investigate the efficacy of combined radiotherapy and sacituzumab govitecan toward ocular metastases.

## 1 Introduction

Orbital metastases are rare in patients with breast cancer [1]. Their presentation can often be asymptomatic, suggesting that this may be an underrepresentation. Autopsy studies have revealed microscopic ocular metastases in approximately 40% of deceased breast cancer patients, rates that align more closely with central nervous system (CNS) metastases observed in nearly 50% of women with advanced triple negative or HER2 positive breast cancer [2, 3]. Treatment of orbital metastases focuses on preserving visual function and palliation of symptoms. Radiation therapy is the primary modality, although it carries considerable potential toxicities including cataract formation and radiation retinopathy [2]. Surgical intervention may be considered for pain relief and management of visual symptoms, but it entails a high risk of ocular morbidity [2].

Medical management of orbital metastases is limited by the inability of treatment options to penetrate the blood brain barrier [4]. As systemic treatment of CNS metastases in breast cancer is dependent on hormone receptor status, options in patients with triple negative breast cancer (TNBC) are further limited [5]. Current chemotherapy treatment options for brain metastases in TNBC include methotrexate, etoposide, cisplatin, capecitabine, adriamycin, cytoxan, and eribulin [4, 5, 6]. Targeted therapies including PARP inhibitors have also been used in TNBC, though current data regarding their efficacy is limited [6, 7]. The efficacy of immunotherapy for brain metastases in TNBC patients is limited, as patients with brain metastases were excluded from initial clinical trials such as KEYNOTE 355 [4, 8].

Sacituzumab govitecan, an antibody drug conjugate, has demonstrated improved overall survival and progression‐free survival over traditional chemotherapy in patients with metastatic TNBC [9, 10]. To our knowledge, there is no prior literature documenting successful treatment of orbital metastases with sacituzumab govitecan. Prior case reports of patients with choroidal metastases due to breast cancer were limited to radiotherapy only [11, 12]. We report the first case of orbital metastases successfully treated with radiation therapy and sacituzumab govitecan.

## 2 Case Presentation

The patient is a 61‐year‐old female with metastatic triple negative breast cancer treated at the Chao Family Comprehensive Cancer Center. She was initially diagnosed in September 2018 with a left breast hormone receptor positive invasive ductal carcinoma. She received neoadjuvant chemotherapy with doxorubicin, cyclophosphamide, and docetaxel. She underwent a partial mastectomy with sentinel lymph node biopsy in March 2019 with pathologic stage T2N1a, hormone receptor positive, HER2 1+. Adjuvant endocrine therapy was recommended, but the patient declined.

She was doing well until March 2022 when she presented with cough. A CT scan was performed and was concerning for a left lower lobe mass with mediastinal and hilar lymphadenopathy. In July 2022, bronchoscopy was performed and was positive for metastatic triple negative breast cancer. Staging scans at that time also showed hepatic, osseous, and right supraclavicular and multistation thoracic nodal metastases. Given symptomatic dyspnea, the patient underwent palliative radiation with 3000 cGy to the L Lung, L hilum in 10 fractions of 300 cGy using the IMRT‐VMAT technique with 6× photons over 13 days from August 1 through August 13, 2022.

Concurrently, the patient noted floaters in her vision on August 15, 2022. She was seen by her optometrist on September 1, 2022, who found bilateral choroidal masses. Ultrasound performed by ophthalmology noted a 4 × 11 mm left orbital mass and a 2 × 10 mm right orbital mass. Radiation to both eyes, along with systemic chemotherapy, was recommended. Radiation therapy was performed in unilateral eyes to ensure the patient tolerated therapy with close monitoring of the contralateral eye for signs of disease progression. From September 2022 to October 7, 2022, she received radiation with 4500 cGy to the left eye in 25 fractions of 180 cGy using the IMRT‐VMAT technique with 6× photons over 36 days and 3420 cGy to the right eye in 19 fractions of 180 cGy using the IMRT‐VMAT technique with 6× photons over 27 days. The patient developed severe dry eyes from radiation keratopathy, which was managed with artificial tears. She was subsequently started on sacituzumab govitecan, receiving cycle 1 on October 5, 2022, and day 8 on October 19, 2022, due to scheduling delays. Ophthalmic exam on October 21, 2022, showed a dramatic decrease in size of bilateral tumors.

The patient completed 6 cycles of sacituzumab govitecan until disease progression in February 2023. While on treatment, the patient experienced intermittent diarrhea and fatigue. MRI brain on February 18, 2023 showed multiple enhancing foci in the bilateral cerebellum and 1.2 cm enhancing pituitary lesion. PET scan on February 21, 2023 showed no other areas of progression. She began treatment with doxorubicin in April 2023. The patient continued to have progression of disease and developed obstructive hydrocephalus and elected to pursue hospice care in May 2023. She passed away in June 2023.

**FIGURE 1:**
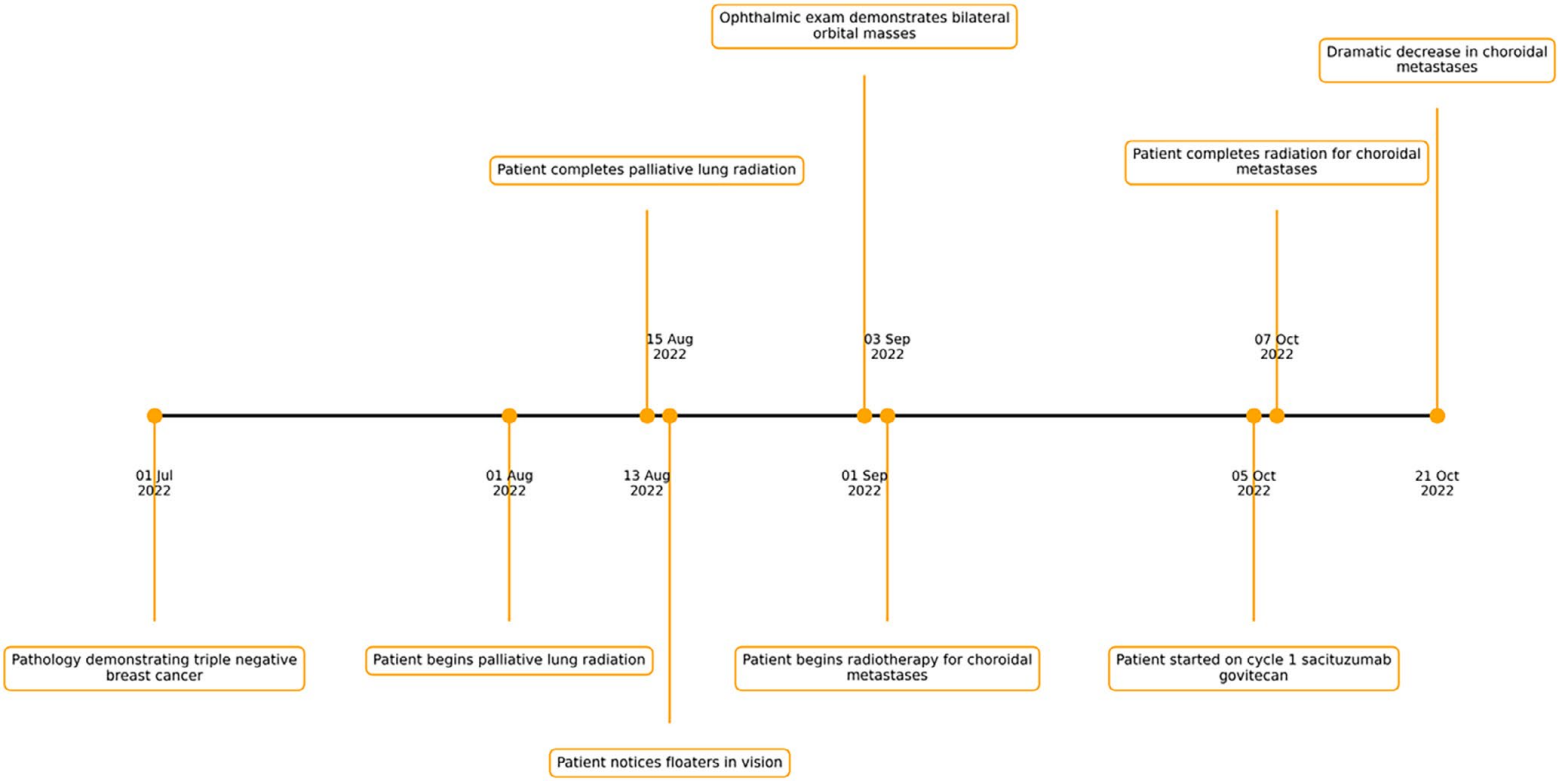
Treatment timeline.

**FIGURE 2:**
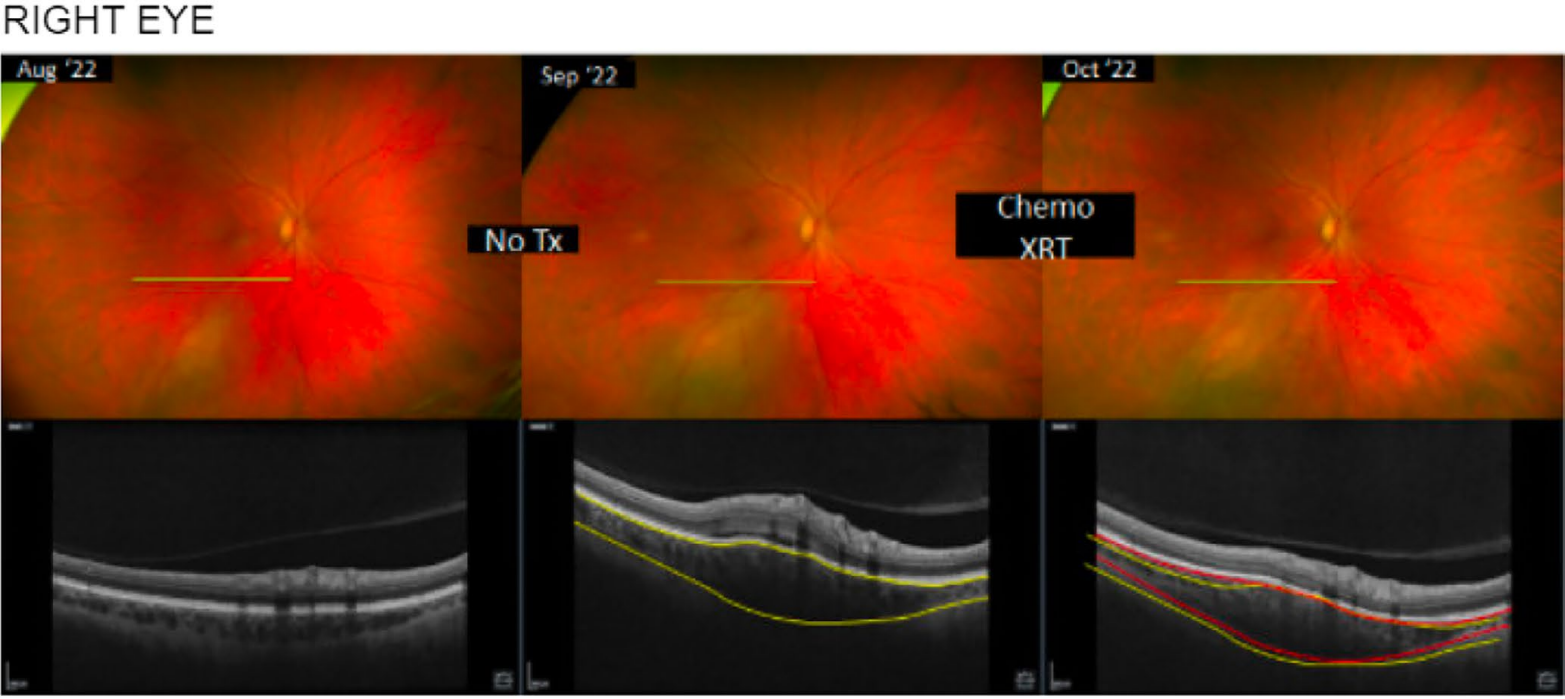
Fundus photography and ocular ultrasound of the right eye demonstrating choroidal metastasis before treatment and dramatic reduction in tumor size with interval development of chorioretinal scar following combination radiotherapy and sacituzumab govitecan.

**FIGURE 3:**
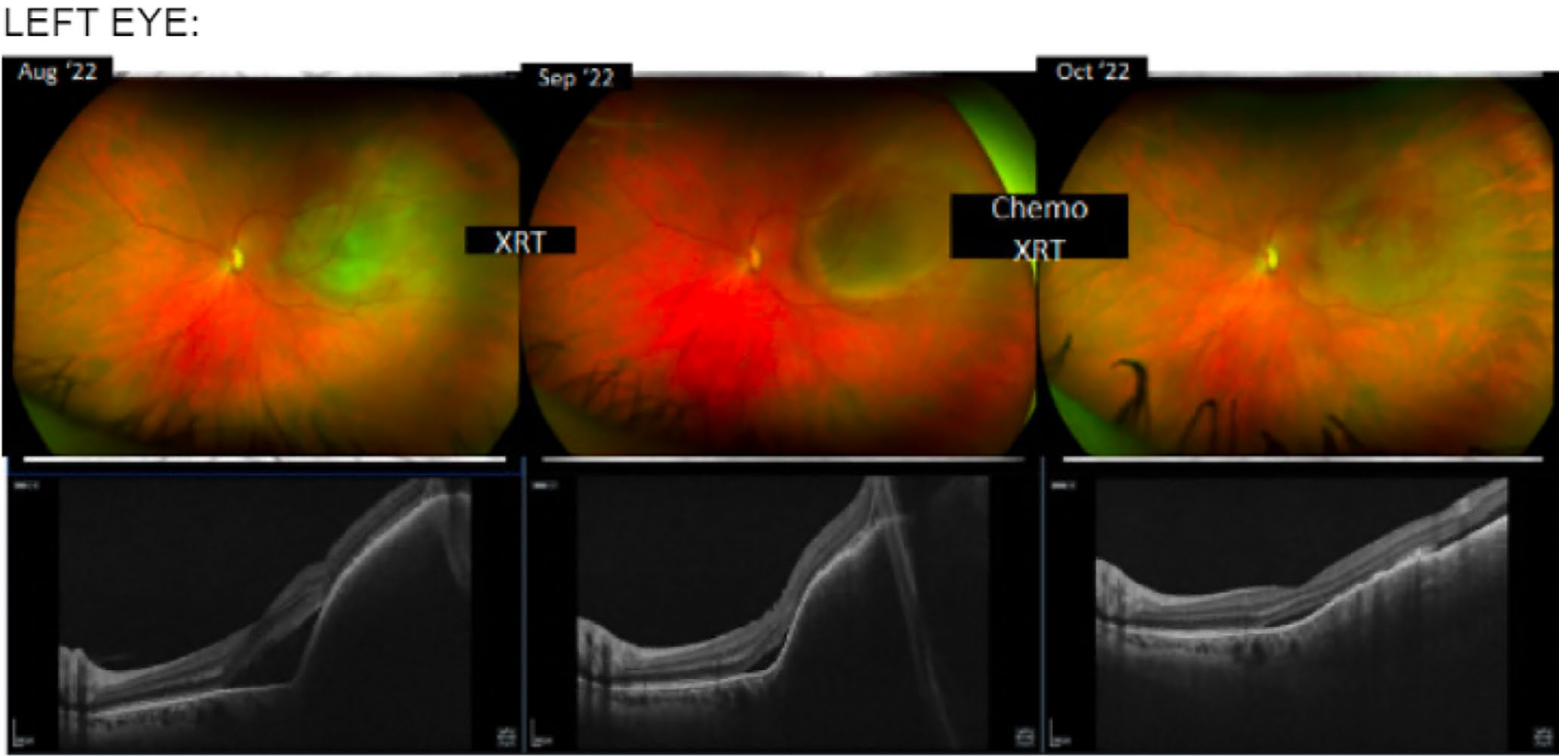
Fundus photography and ocular ultrasound of the left eye demonstrating choroidal metastasis before treatment and dramatic reduction in tumor size with interval development of chorioretinal scar following combination radiotherapy and sacituzumab govitecan.

Our patient's timeline is reported in Figure 1 and ophthalmologic evaluation is reported in Figures 2 and 3.

Treatment timeline detailing key points in the treatment course including cancer diagnosis, timing of XRT, onset of orbital metastases, radiotherapy, and systemic therapy with sacituzumab govitecan.

## 3 Discussion

Following the results of the ASCENT trial, which demonstrated a significant improvement in survival among patients with triple‐negative breast cancer, sacituzumab govitecan received initial approval for use in patients with metastatic breast cancer who had previously undergone at least two systemic therapies—one of which must have been administered in the metastatic setting [9, 13]. This approval marked a meaningful advancement in the treatment landscape for a population with limited options. Patients with brain metastases were excluded from primary endpoints; however, a subgroup analysis of included patients with stable brain metastases was performed. Overall, 61 of 529 (12%) enrolled patients had stable brain metastases at screening and were randomized to sacituzumab govitecan (*n* = 32) or single‐agent treatment of physician's choice (*n* = 29) [10, 14]. In this subset, median PFS was 2.8 months (95% CI, 1.5–3.9) for sacituzumab govitecan versus 1.6 months (95% CI, 1.3–2.9) for single‐agent treatment of physician's choice, and no significant improvement in OS was seen [14].

To date, case reports of choroidal metastases in patients with triple‐negative breast cancer are exceedingly limited with targeted radiation as a mainstay of therapy (Table 1) [6]. Ladiratuzumab vedotin, an antibody‐drug conjugate that targets the zinc transporter and microtubules, has shown efficacy in metastatic TNBC [19, 20, 21]. However, the activity of ladiratuzumab vedotin against brain metastases and CNS disease is unclear, as prior clinical trials have required treatment of brain lesions prior to enrollment [22, 23]. Trastuzumab deruxtecan has demonstrated significant improvements in progression‐free survival in metastatic TNBC [21, 24]. In patients with brain metastases, trastuzumab deruxtecan has shown comparable outcomes compared to patients with extracranial only disease [25, 26]. In contrast, subgroup analyses of the phase III ASCENT trial of patients with metastatic TNBC and brain metastases treated with sacituzumab govitecan found small improvements in PFS compared to single‐agent chemotherapy that did not meet statistical significance [14]. Similarly, a multicenter study of patients with metastatic TNBC and active brain metastases found an intracranial response rate of 42% [27]. Case reports of patients with TNBC receiving sacituzumab govitecan have demonstrated promising disease response to both brain metastases and leptomeningeal carcinomatosis [28, 29]. This case presentation adds to a limited body of literature demonstrating the effectiveness of sacituzumab govitecan toward CNS disease in TNBC.

**TABLE 1:** Systemic therapies for choroidal metastases in breast cancer.

Class of systemic therapy	Example agents	Advantages	Limitations	References
Endocrine therapies	Tamoxifen, aromatase inhibitors	Non‐cytotoxic, well tolerated in many patients	Slow‐onset, not effective for hormone‐resistant disease	[6]
Cytotoxic chemotherapy	Platinum agents, taxanes, anthracyclines	Treats both ocular and extraocular disease	Variable ocular penetration; systemic toxicities; response depends on chemosensitivity of primary tumor	[4, 5, 6, 15]
HER2‐targeted therapy	Trastuzumab, pertuzumab, lapatinib	Can produce rapid, durable ocular responses in HER2‐driven disease	Cardiotoxicity (trastuzumab ± anthracyclines), variable ocular penetration for large antibodies (TKIs may penetrate better)	[16, 17]
Immune checkpoint inhibitors	Anti‐PD‐1: pembrolizumab, nivolumab	Potential for durable systemic and ocular control in responsive tumors; may avoid local radiation in some responders	Responses may be delayed; risk of immune‐related ocular inflammation (uveitis, serous detachments)	[4, 8, 18]

Ultimately, this case highlights the importance of sacituzumab govitecan as a potential option for TNBC patients with brain metastases. The ongoing S2007 trial aims to shed further light on the efficacy of sacituzumab govitecan in TNBC patients with CNS disease [30]. While at least 1 systemic therapy is typically warranted in patients with metastatic disease prior to starting sacituzumab govitecan, this case report and promising data regarding CNS metastases suggest that sacituzumab govitecan should be considered earlier in a patient's treatment course [13]. Furthermore, prior studies have demonstrated that a longer time to develop CNS metastases is considered a positive prognostic factor [31, 32]. Taken with data demonstrating that sacituzumab govitecan prolongs progression‐free survival, this further underscores our assertion that this promising antibody‐drug conjugate should be considered for earlier use in patients with metastatic TNBC [9, 33].

This case report has a few limitations. Given that the patient received radiation prior to sacituzumab govitecan, we are unable to attribute the patient's response to orbital metastases entirely to her systemic treatment. It does, however, highlight the efficacy, safety, and tolerability of combination radiation and sacituzumab govitecan. Furthermore, as a single case without direct comparators, we are unable to generalize this to all patients with metastatic TNBC and ocular metastases. Further studies are needed to investigate the efficacy of combined radiotherapy and sacituzumab govitecan toward ocular metastases.

## 4 Conclusion

Management of CNS disease in metastatic TNBC is limited by lack of targeted therapies and lack of penetration of the blood brain barrier by systemic treatments. Limited studies and case reports to date suggest that sacituzumab govitecan may have utility for patients with TNBC and intracranial involvement. To date, case reports of choroidal metastases in patients with TNBC are exceedingly limited. In this case report, we highlight an effective and tolerable treatment of combination radiation therapy with systemic sacituzumab govitecan for CNS disease in metastatic TNBC. While radiation therapy is typically first line for choroidal metastases, our findings suggest that sacituzumab govitecan may hold value as part of a multimodal regimen for ocular metastases in TNBC. Further studies and real‐world data are needed to investigate the efficacy of combined radiotherapy and sacituzumab govitecan toward ocular metastases.

## Author Contributions

**Nellie Nafissi:** conceptualization, investigation, writing – original draft, writing – review and editing, project administration, supervision. **Aditya Mahadevan:** conceptualization, investigation, writing – original draft, writing – review and editing. **Elaine Chiao:** investigation, writing – original draft, writing – review and editing. **Nejina Rijal:** investigation, writing – original draft, writing – review and editing. **Ritesh Parajuli:** conceptualization, investigation, writing – original draft, writing – review and editing, project administration, supervision.

## Funding

The authors have nothing to report.

## Consent

Informed consent was obtained from the patient regarding the publication of this case and the use of images pertaining to this case.

## Conflicts of Interest

The authors declare no conflicts of interest.

## Data Availability

Data sharing not applicable to this article as no datasets were generated or analyzed during the current study.
